# Cognitive Profile of Children and its Relationship With Academic Performance

**DOI:** 10.32598/bcn.9.10.230

**Published:** 2019-03-01

**Authors:** Abbas Nesayan, Malahat Amani, Roghayeh Asadi Gandomani

**Affiliations:** 1. Department of Psychology, Faculty of Humanities, University of Bojnord, Bojnord, North Khorasan, Iran.

**Keywords:** Cognitive profile, Children, Academic Performance

## Abstract

**Introduction:**

Cognitive abilities are necessary for successful learning. Children with different cognitive ability levels may have diverse performances. The current study aimed at investigating the cognitive profile in children and its relationship with Academic Performance.

**Methods:**

The population of the current cross sectional study consisted of all students in Jajarm City, Iran. The subjects were selected using multi-stage cluster sampling; and 289 students aged 6 to 13 years were included in the study. Data were collected using the Rey-Osterrieth complex figure test, coding subtest from Wechsler intelligence scale and behavioral rating inventory of executive functions. The Spearman rank correlation coefficient and the Scheffe post hoc test were used for data analysis.

**Results:**

The obtained results showed that ‘processing speed’, ‘perceptual organizational ability’, ‘monitoring’, ‘planning’, ‘working memory’, ‘initiate’, ‘emotional regulation’, ‘shifting’, and ‘inhibit ‘ were significantly correlated with Academic Performance (P<0.001).

**Conclusion:**

There was a significant relationship between cognitive profile and Academic Performance. Thus, teachers need to consider the variable of cognitive abilities in academic success.

## Highlights

- One of the most important capabilities of children is their cognitive abilities.
- Cognitive abilities can affect many children's functions such as individual, educational, and social activities.
- Cognitive abilities have a significant relationship with academic performance in children.

## Plain Language Summary

Children’s entering school can be one of the most challenging events in their life. Different factors can affect how children perform in school. Many researchers focus on social and economic factors that can predict academic success, but there are other abilities that affect learning and understanding in children; one of these is executive functions. Executive functions include skills such as working memory, set shifting, emotion regulation, organization, monitoring, and controlling. Executive functions help children learn abstract concepts, participate in classwork, and regulate their emotion. The results of this study showed that cognitive abilities could well predict academic performance. Children who are more powerful in executive functions tasks achieve higher academic performance.

## 1. Introduction

One of the major developmental periods is the transition from early childhood to formal education (Duncan, McClelland, & Acock, 2017), which can be particularly challenging due to cognitive abilities, learning context, and various individual experiences (Lo, Chen, & Lin, 2017). In this period, academic achievement exerts a huge impact on self-concept, motivation, and diligence of children (Jayanthi et al., 2014). The children’s success in educational contexts is a variable of certain factors such as the ability to deal with conceptual and abstract problems and critical thinking (Luong et al., 2017). There is growing interest in determining the factors impressing Academic Performance. Many findings documented the influence of social and contextual factors. The socioeconomic status is one of the factors that can indirectly affect the educational achievement of children.

Researchers also examine the effects of children’s characteristics, focusing on how they learn; rather than studying the specific areas of knowledge (such as letters or numbers), they examine general mental processes (Nesbitt, Baker-Ward, & Willoughby, 2013). In this case, executive functions help students to take advantage of educational opportunities in classrooms (Duncan et al., 2017). Executive function can play an pivotal role in successful learning in future (Brock et al., 2009).

Despite the uncertainties surrounding the nature of executive functions (Best, Miller, & Naglieri, 2011), they can be defined as the basic cognitive abilities underlying planning, flexibility, self-regulation, and purposeful behavior (Munro et al., 2017). Executive functions represent the abilities that organize, order, and handle necessary information for daily activities (McCloskey, 2015). Executive functions commonly coordinate a higher level of thinking processes, which lays the foundation for problem-solving ability, and are vital for conditions that call for active control over thoughts and actions (Brock et al., 2009). Executive function is defined as a multidimensional structure that facilitates cognitive regulation (Nesbitt et al., 2013). Although the constructive components of executive functions are highly interrelated, they are often defined as separate components (Becker et al., 2014). Executive functions include skills such as working memory, set shifting, and inhibit controlling (Baptista et al., 2016; Becker et al., 2014; Duncan et al., 2017; Verdejo-Garcia & Manning, 2015).

The working memory allows the retention of information in mind for a period of time. This is an essential component for successful completion of assignments (Lan et al., 2011). Self-regulation is a critical factor frequently considered by teachers to assess student’s performance. As far as executive functions are concerned, self-regulation can be reflected in areas such as proper pacing and planning of tasks under certain time limitation (McCloskey, 2015). Attention shifting is the ability to change activities based on situational requests. Inhibition refers to the ability to control the response or ignore the information that impedes the completion of tasks (Nesbitt et al., 2013). Studies reveal that executive functions are traditionally linked to the prefrontal cortex (Becker et al., 2014; Moriguchi & Hiraki, 2013) but recent studies suggest the involvement of other brain areas including parietal lobes, temporal lobes, and cerebellum (Munro et al., 2017).

In the past, many studies examined executive function in children and its relationship with different variables such as functional outcomes (Bull & Lee, 2014), academic readiness (Baptista et al., 2016), social-behavioral functioning (Diamantopoulou et al., 2007), behavioral regulation (Duncan et al., 2017), mathematics achievement (Blankson & Blair, 2016; Cragg et al., 2017; Dulaney, Vasilyeva, & O’Dwyer, 2015), visuomotor skills (Becker et al., 2014), reading comprehension (García-Madruga et al., 2014), and problematic behaviors (Munro et al., 2017). Academic Performance is a factor often considered in relation to executive functions (Best et al., 2011; Brock et al., 2009; Lan et al., 2011). Children without poor executive function have trouble controlling impulsive behaviors and regulating their emotions, which hinder their participation in the classroom activities and subsequently affect their Academic Performance (Baptista et al., 2016).

In the classroom, children should be able to use executive functions to shift between assignments, follow orders, and communicate with peers. For example, when a child goes from playground to the math class, s/he should be able to inhibit the desire to continue playing, listen to the teacher’s instructions, retain them in the mind and start a new activity (Becker et al., 2014). When working on abstract concepts, children should be able to use executive functions or cognitive problem solving. Strong working memory, inhibition, and attention capabilities can help children on their path to success. Children should be able to recall instructions and lessons in the classroom (working memory) and concentrate on the important features of the learning environment (attention). In addition, they should be able to stay on an assignment for a certain period of time (impulse control). In this respect, executive functions constitute an essential component for the student’s progress (Brock et al., 2009).

The results of studies on Academic Performance are heterogeneous, with most of these studies focusing only on some aspects of executive functions. Brock et al. (2009) studied hot and cold executive functions, math achievement and learning behavior in kindergarten children, finding that executive functions predicted math progress and learning-related behaviors, but none of these variables were predicted by hot executive functions. Lan et al. (2011) demonstrated that working memory, inhibit, and attentional control were predictors of academic achievement in preschool children. Best et al. (2011), measured complex performance functions such as completion time and accuracy in children aged 5 to 17 years, suggesting that the relationship between components of executive function and academic domains was a variable of the age.

Two types of cognitive function assessment were used in the current study: tasks that directly assessed cognitive functions (coding and the Rey-Osterrieth complex figure test) and Behavioral Rating Inventory of Executive Function (BRIEF) filled out by teachers. Multiple evaluation methods were utilized to gain deeper insights into children’s cognitive abilities in the learning environment. BRIEF is a comprehensive tool used to measure eight executive function subscales including initiating, working memory, planning/organizing, organizing the materials, monitoring (metacognition scale), inhibit, shifting, and emotional controlling (behavioral regulation scale) (Toplak, West, & Stanovich, 2013). The current study aimed at examining cognitive profile in children and its relationship with Academic Performance.

## 2. Methods

### 2.1. Participants

The population of the current cross sectional study consisted of all children aged 6 to 13 years in Jajarm City, Iran. The subjects were selected using multistage cluster sampling method and 289 students aged 6 to 13 years along with their teachers were include in the study; 49.1% of participants were male and 51.9% female. The inclusion criteria were no history of physical and neurological diseases or developmental disorders and an age range of 6 to 13 years.

### 2.2. Procedure

All of the participants took the coding and Rey-Osterrieth complex figure test (A card). First, Rey-Osterrieth complex figure was presented to the subjects and then they were asked to take the coding test. In the next step, teachers completed the BRIEF questionnaire for each student. The researchers explained to teachers how to complete the questionnaire. However, they were not aware of the purpose of the study. The Academic Performance of children was described in four categories (poor=D, moderate=C, good=B, and very good=A) based on their educational records. The data analysis was performed with SPSS V. 19. Statistical indices such as mean, standard deviation, the Spearman rank correlation coefficient and the Scheffe post hoc test were used for data analysis.

### 2.3. Measures

#### 2.3.1. The Rey-Osterrieth complex figure test

The Rey-Osterrieth complex figure test was first developed by Andre Rey in 1941 and then standardized by Paul-Alex Osterrieth in 1944 (Dimitrov et al., 2015). This test assesses visuospatial abilities, attention, and executive function (Dimitrov et al., 2015), perceptual organization (Fastenau, Denburg, & Hufford, 1999), visuoconstructional abilities (Frank & Landeira-Fernandez, 2008) and visual and nonverbal memory (Dimitrov et al., 2015; Fastenau et al., 1999; Frank & Landeira-Fernandez, 2008). It consists of two parts. The first part assesses the perceptual organization and the second part measures visual memory. To administer the Rey-Osterrieth complex figure test, participants are asked to reproduce a complex figure (containing 18 components) on a sheet as accurately as possible (Dimitrov et al., 2015; Fastenau et al., 1999; Frank & Landeira-Fernandez, 2008). After 30 minutes, they are instructed to draw what they recall on a sheet (Dimitrov et al., 2015; Fastenau et al., 1999; Frank & Landeira-Fernandez, 2008).

#### 2.3.2. Coding subtest from the Wechsler intelligence scale for children

Coding test (the digit symbol) is one of subtests of the Wechsler intelligence scale (Crowe et al., 1999). It is used to measure perceptual-motor speed (Ebaid et al., 2017; Joy, Kaplan, & Fein, 2004), processing speed (Bachman et al., 2010; Ebaid et al., 2017; González-Blanch et al., 2010), and executive functions (González-Blanch et al., 2010). In this task, the subject is required to scan stimuli efficiently so that s/he can generate the correct response quickly and accurately (Rodgers et al., 1999). The task contains rows of blank squares that are randomly numbered from 1 to 9. At the top of the page, there is an image where each number (1 to 9) matches a specific symbol. The subject has 120 seconds to pair numbers with corresponding symbols according to the provided images (Crowe et al., 1999; González-Blanch et al., 2010). The subject’s score is computed based on the number of symbols correctly identified (Ebaid et al., 2017).

#### 2.3.3. The behavioral rating inventory of executive functions

One of the most commonly used tools to measure executive functions is the Behavioral Rating Inventory of Executive Functions (BRIEF) (Toplak et al., 2013) that assesses behavioral manifestations of executive functions in children aged 5 to 18 years (Anderson et al., 2009; Gioia & Isquith, 2004). The BRIEF developed by Gioia et al. (2000) consists of two indexes: metacognition and behavioral regulation. Metacognition is the ability to track information and monitor actions during daily activities and behavioral regulation measures self-regulation and proper behavior (Anderson et al., 2009; McAuley et al., 2010).

It assesses eight subdomains of executive functions: inhibit, shift (flexibility), emotional control, initiate, working memory, plan-organize, organization of materials, and monitor (Anderson et al., 2009; Gioiaet al., 2002; Toplak et al., 2013). This questionnaire has two different forms, one completed by the teacher and one by parents (Mangeot et al., 2002). In the current study, the teachers’ form was used. The Cronbach’s alphas for teachers` form were as follows: global executive composite α=0.97; inhibit α=0.94; shift α=0.90; emotional control α=0.91; working memory α=0.94; plan/organize α=0.97. The results of test-retest reliability for this form were as follows: global executive composite, r=0.88; inhibit, r=0.94; shift, r=0.65; emotional control, r=0.83; working memory, r=0.88; and plan/organize, r=0.85 (Isquith et al., 2005).

## 3. Results

The current study comprised of 289 children in the age range of 6 to 13 years (Mean=9.59, SD=1.64). There were 139 male and 144 female subjects in the study. Tables 1 and 2 show demographic information of the participants. As shown in Table 3, there was a significant relationship between cognitive profile and Academic Performance. Table 4 indicates the results of ANOVA for the cognitive profile at different levels of Academic Performance.

**Table 1.** Demographic information of the participants

**Variable**	**N**	**Minimum**	**Maximum**	**Mean** ±**SD**
Age	283	6.90	13	9.59±1.64

**Table 2.** Demographic information of the participants

**Variables**	**N**	**Percent**	**Cumulative Percent**
Male	139	49.1	49.1
Female	144	50.9	100
Total	283	100	

**Table 3.** Relationship between cognitive profile and academic achievement

**Variables**	**1**	**2**	**3**	**4**	**5**	**6**	**7**	**8**	**9**	**10**	**11**	**12**	**13**	**14**
Academic achievement	1 ^*^													
Coding	0.17 ^**^	1												
Ray	0.22 ^*^	-	1											
Inhibit	−0.70 ^*^	-	-	1										
Shift	−0.68 ^*^	-	-	-	1									
Emotion	−0.64 ^*^	-	-	-	-	1								
Initial	−0.59 ^*^	-	-	-	-	-	1							
Working memory	−0.64 ^*^	-	-	-	-	-	-	1						
Planning	−0.73 ^*^	-	-	-	-	-	-	-	1					
Organization	−0.59 ^*^	-	-	-	-	-	-	-	-	1				
Monitor	−0.65 ^*^	-	-	-	-	-	-	-	-	-	1			
Br	−0.72 ^*^	-	-	-	-	-	-	-	-	-	-	1		
MI	−0.69 ^*^	-	-	-	-	-	-	-	-	-	-	-	1	
GEC	−0.71 ^*^	-	-	-	-	-	-	-	-	-	-	-	-	1

* Correlation is significant at the 0.01 level;

** Correlation is significant at the 0.05 level.

**Table 4.** Results of ANOVA of cognitive profile in Academic Performance levels

**Variables**		**Sum of Squares**	**df**	**Mean Square**	**F**	**Sig.**
GEC	Intergroup	142264.52	3	47421.50	104.500	0.000
	Intragroup	126608	279	453.79		
MI	Intergroup	53180.58	3	17726.86	92.31	0.000
	Intragroup	53576.65	279	192.03		
BRI	Intergroup	21053.63	3	7017.87	107.35	0.000
	Intragroup	18238.81	279	65.37		
Monitor	Intergroup	1982.82	3	660.94	72.07	0.000
	Intragroup	2558.33	279	9.170		
Organization	Intergroup	800.96	3	266.98	56.37	0.000
	Intragroup	1321.43	279	4.73		
Planning	Intergroup	5625.17	3	1875.05	113.56	0.000
	Intragroup	4606.55	279	16.51		
Working memory	Intergroup	2356.71	3	785.57	67.56	0.000
	Intragroup	3244	279	11.62		
Initiate	Intergroup	1319.11	3	439.70	60.39	0.000
	Intragroup	2031.31	279	7.28		
Emotional	Intergroup	1993.83	3	664.61	66.57	0.000
	Intragroup	2785.11	279	9.98		
Shift	Intergroup	1965.46	3	655.15	86.11	0.000
	Intragroup	2122.73	279	7.60		
Inhibit	Intergroup	3956.98	3	1318.99	97.36	0.000
	Intragroup	3779.58	279			
Coding	Intergroup	4309.41	3	1436.47	7.35	0.000
	Intragroup	54523.40	279	195.42		
Ray	Intergroup	325.82	3	108.60	6.42	0.000
	Intragroup	4718.81	279	16.91		

As shown in Table 4, there was a significant difference between the levels of Academic Performance in cognitive profile; the Scheffe post hoc test was used to determine the significant difference between the different levels (Table 5). Children with poor Academic Performance compared with those of other levels of performance (moderate, good and very good) obtained a lower score in cognitive profile.

**Table 5.** Scheffe Post Hoc Test for multiple comparisons between Academic Performance levels

**Dependent Variable**	**I**	**J**	**Mean Difference I–J**	**Std. Error**	**Sig.**	**95% CI**	

						**Lower**	**Upper**
GEC	D	C	31.63	5	0.000	17.56	45.69
		B	56.08	4.82	0.000	42.52	69.65
		A	71.61	4.61	0.000	58.64	84.58
MI	D	C	20.35	3.25	0.000	11.20	29.50
		B	35.32	3.13	0.000	26.49	44.14
		A	44.18	2.99	0.000	35.74	52.62
BRI	D	C	11.77	1.89	0.000	6.44	17.11
		B	20.76	1.83	0.000	15.61	25.91
		A	27.43	1.75	0.000	22.50	32.35
Monitor	D	C	3.70	0.71	0.000	1.70	5.69
		B	6.61	0.68	0.000	4.68	8.54
		A	8.43	0.65	0.000	6.59	10.28
Organization	D	C	2.85	0.51	0.000	1.41	4.29
		B	4.53	0.49	0.000	3.15	5.92
		A	5.57	0.47	0.000	4.24	6.89
Planning	D	C	5.94	0.95	0.000	3.25	8.62
		B	11.15	0.92	0.000	8.56	13.74
		A	14.04	0.87	0.000	11.57	16.52
Working memory	D	C	3.61	0.80	0.000	1.36	5.86
		B	6.73	0.77	0.000	4.55	8.90
		A	9.02	0.73	0.000	6.94	11.09
Initial	D	C	3.77	0.63	0.000	1.99	5.55
		B	6.28	0.61	0.000	4.56	8
		A	7.10	0.58	0.000	5.46	8.74
Emotional	D	C	3.80	0.74	0.000	1.71	5.88
		B	6.34	0.71	0.000	4.33	8.35
		A	8.53	0.68	0.000	6.60	10.45
Shift	D	C	3.64	0.64	0.000	1.82	5.46
		B	6.17	0.62	0.000	4.42	7.93
		A	8.41	0.59	0.000	6.73	10.09
Inhabit	D	C	4.91	0.86	0.000	2.47	7.34
		B	9.26	0.83	0.000	6.92	11.61
		A	11.75	0.79	0.000	9.51	13.99
Coding	D	C	13.77	3.28	0.001	23.00	4.54
		B	12.24	3.16	0.002	21.14	3.33
		A	13.77	3.02	0.000	22.28	5.26
Ray	D	C	2.68	0.96	0.054	5.39	0.03
		B	2.71	0.93	0.038	5.33	0.09
		A	3.82	0.89	0.000	6.32	1.32

## 4. Discussion

Academic Performance is strongly associated with future achievements. People who are more successful at school are more likely to be recruited in stable jobs with higher earnings. Therefore, determining the factors associated with Academic Performance is of paramount importance. The current study aimed at investigating cognitive profile in children and its relationship with Academic Performance.

Consistent with the current study results, Dulaney et al. (2015) showed that children with weak attention span and short-term memory had lower academic achievement. In another study, García-Madruga et al. (2014) found that working memory could predict changes in academic achievement. Also, Baker et al. (2014), observed that only inhibition and working memory functions were associated with academic achievement. However, the study by Brock et al. (2009) revealed that hot executive function did not anticipate learning-related behaviors and academic achievement. Vandenbroucke, Verschueren, & Baeyens et al. (2017) indicated that working memory was the major predictor of academic achievement while cognitive flexibility played a restricted role and inhibition was not correlated with academic achievement.

The current study results showed that all cognitive components and Academic Performances were related. Among cognitive components, processing speed (coding) and perceptual organizational ability (Rey test) were weakly correlated with Academic Performance. Coding and Ray tests were directly administered to the participants but other cognitive abilities were completed by the teachers. A teacher’s attitude about the ability of students may affect how they answer the questionnaire.

Cognitive abilities such as impulse controlling, planning, and monitoring are crucial for both areas of learning (reading and mathematics) (Best et al., 2011). Executive function skills can help the development of academic standards in children as they provide apt opportunities for learning. Children that can concentrate on the learning content, retain information components in the mind, and deal with challenges are more successful in the academic environment (Duncan et al., 2017).

Cognitive abilities are essential for the success of children at school. For example, when an image is presented to a child as a task, s/he should be able to stay focused, concentrate on the information, and use his/her ability to control the impulses before complete processing of the image. In addition, s/he should retain all that information in memory. When the child goes from one image to another, s/he should be able to shift attention (Becker et al., 2014).The current study had several limitations that should be considered in future studies; emotional factors such as anxiety that can affect executive functions and Academic Performance in children were not considered. It is recommended that future studies consider such variables. The current study had a limited sample size and cautions should be made in generalizing its results to other communities.

The results of the current study indicated a significant relationship between cognitive profile and Academic Performance. These findings suggested that early assessment of cognitive abilities, especially executive functions, can help to identify children at risk of poor Academic Performance. Therefore, early interventions can be offered to help such children before irreparable damages are made. The findings also suggested that executive function training can improve Academic Performance.

## Ethical Considerations

### Compliance with ethical guidelines

Parental consent was obtained for children to participate in the study.
